# A Rare Case of Pyogenic Granuloma on the Lip in a 10-Year-Old Girl: Case Report and Literature Review

**DOI:** 10.7759/cureus.115803

**Published:** 2026-09-04

**Authors:** Madhvika Patidar, Anuradha Agrawal, Parul Jain, Kundendu A Bishen, Hage Monju, Prerna R Batham

**Affiliations:** 1 Department of Oral Pathology and Microbiology, Government College of Dentistry, Indore, IND; 2 Department of Paediatric and Preventive Dentistry, Government College of Dentistry, Indore, IND; 3 Department of Periodontology, Government College of Dentistry, Indore, IND; 4 Department of Orthodontics and Dentofacial Orthopaedics, Government College of Dentistry, Indore, IND

**Keywords:** children, diode laser, granuloma pyogenicum, granuloma telangiecticum, hemangioma, lip, lobular capillary, lobular capillary hemangioma, oral mucosa, pyogenic granuloma

## Abstract

Oral pyogenic granulomas (PGs) are well documented in the literature and are a commonly encountered lesion in routine clinical practice. However, their extragingival occurrence is rare and often has an unusual presentation. The clinical diagnosis of uncommon extragingival PG can be quite challenging. In the present case report, a 10-year-old female presented with a soft, tender swelling of 2 × 1.5 centimetres, which was pink, smooth-surfaced, sessile, and prone to bleeding and was provisionally diagnosed as PG. The excision was performed using a minimally invasive treatment modality of 980-nm diode laser in pulsed mode at 1.5 W under local anesthesia. Histopathological examination confirmed PG. At 3-month follow-up, complete healing was observed without scarring or recurrence. The purpose of this article is to report an unusual case of PG occurring on the lower lip. A further aim is to present a review of the total number of PG of lip cases reported in children through searching databases, citation tracking, and manual reference screening. Very few cases of PG have been reported earlier on the lip in children (up to 14 years).

## Introduction

Pyogenic granuloma (PG) is a commonly occurring inflammatory, single or multiple, proliferative, benign, non-neoplastic, tumour-like, reactive, mucocutaneous lesion of the oral cavity [1,2,3]. The term "pyogenic granuloma" is misleading, as this condition is neither associated with infection nor does it histologically represent a granuloma [4]. The exact etiology of PG is unknown, but there are several associated factors that include low-grade local irritation or trauma, hormonal imbalance, use of various drugs, and poor oral hygiene [5]. It can be sessile or pedunclated, exophytic, erythematous, painless or painful papule or nodule with a smooth or lobulated surface which bleeds spontaneously or on manipulation [2]. PG constitutes 1.8% of all oral pathologies, and 50.35% of reactive lesions [1]. It is the second most common localized reactive hyperplastic lesion of the gingiva after peripheral ossifying fibroma [6]. It was reported in a study that 83% of PG was found on the gingiva, 5.3% on the tongue and lips, 4.2% on the hard palate, 0.8% on the buccal mucosa, and 0.4% on the floor of the mouth [7]. The diagnosis is confirmed by excision biopsy of the lesion followed by histopathological examination [4]. Although PG is a commonly encountered lesion in routine clinical practice, the present case describes a rare extragingival occurrence involving the lower lip in a pediatric patient. The unusual anatomical location and young age of the patient posed a diagnostic challenge, highlighting the importance of considering PG in the differential diagnosis of soft-tissue lesions on the lips.

## Case presentation

A 10-year-old female patient presented to the department with the chief complaint of swelling and bleeding on the lower right side of the lip region for three months. The lesion was initially small and gradually increased in size to its present dimensions of 2 × 1.5 cm (Figure 1).

**Figure 1:**
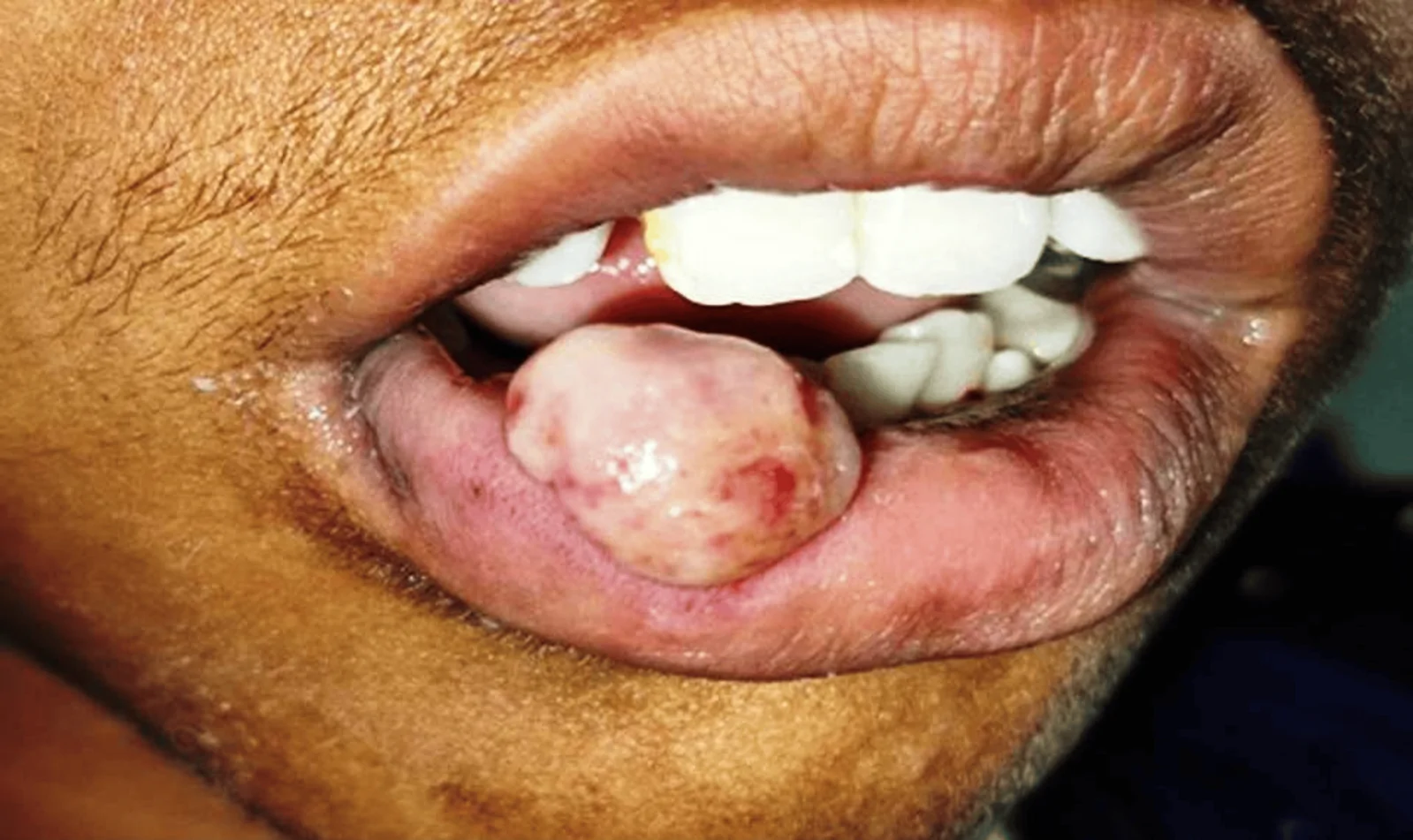
Pre-operative photograph.

On examination, the swelling was exophytic, sessile, pink in colour with a smooth surface. On palpation, the lesion was tender, soft in consistency, and had a tendency to bleed. Correlating clinically, a provisional diagnosis of PG was made, and excisional biopsy was planned. Routine blood investigations were performed, and it was found to be normal. As the patient was young and the lesion showed a bleeding tendency, a diode laser was chosen with a 980-nm pulse wave mode and a power of 1.5W as a minimally invasive procedure (Figure 2).

**Figure 2:**
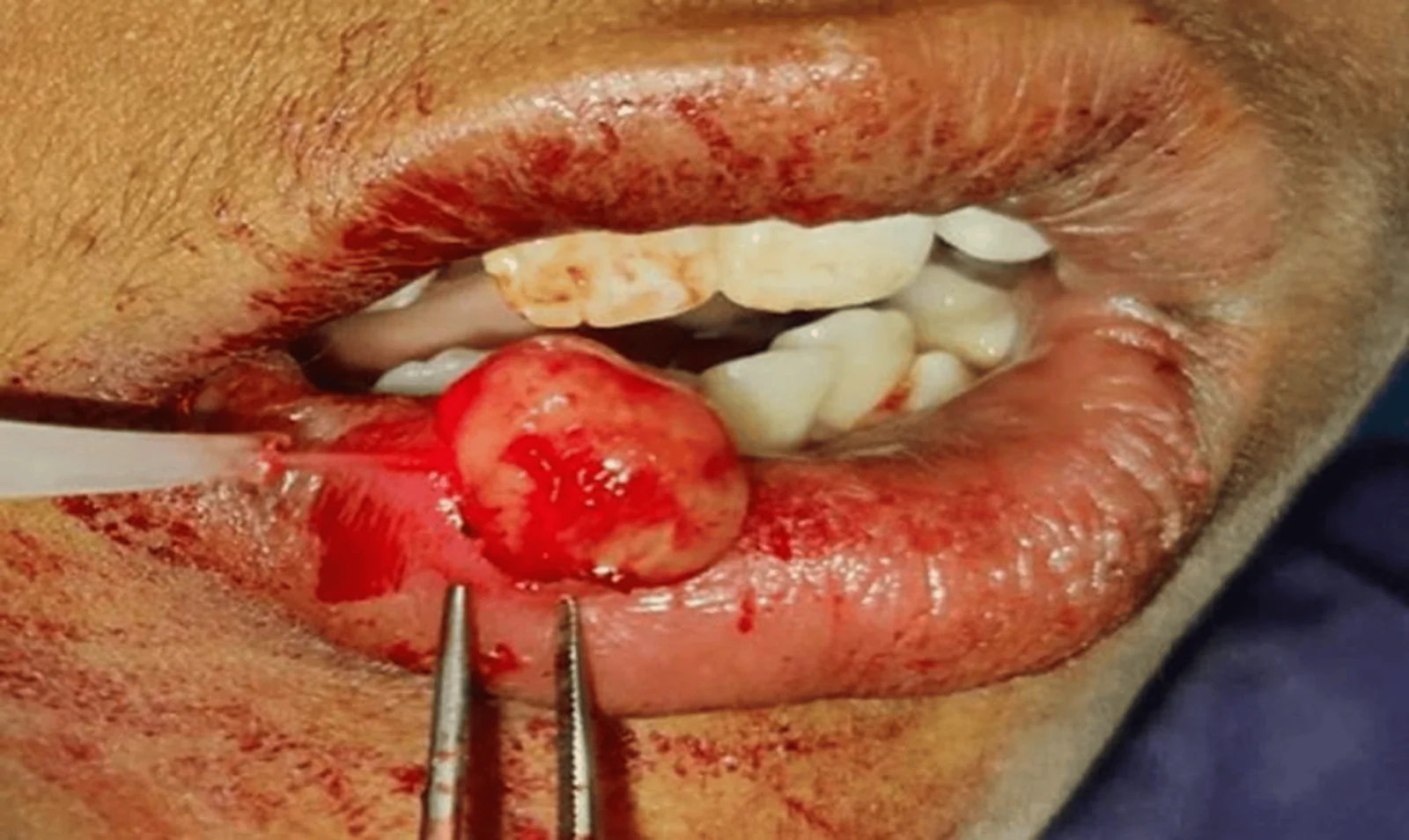
Intra-operative photograph.

After obtaining informed consent from the guardian, the lesion was excised under local anaesthesia (4% articaine with epinephrine in the ratio 1: 200,000). Postoperative instructions were given to the patient and the family. Histopathological examination revealed stratified squamous epithelium showing few areas of ulceration and underlying connective tissue. The connective tissue showed increased vascularity, capillary proliferation, congestion of vessels, and intense diffuse chronic inflammatory cell infiltrate suggestive of PG (Figure 3).

**Figure 3:**
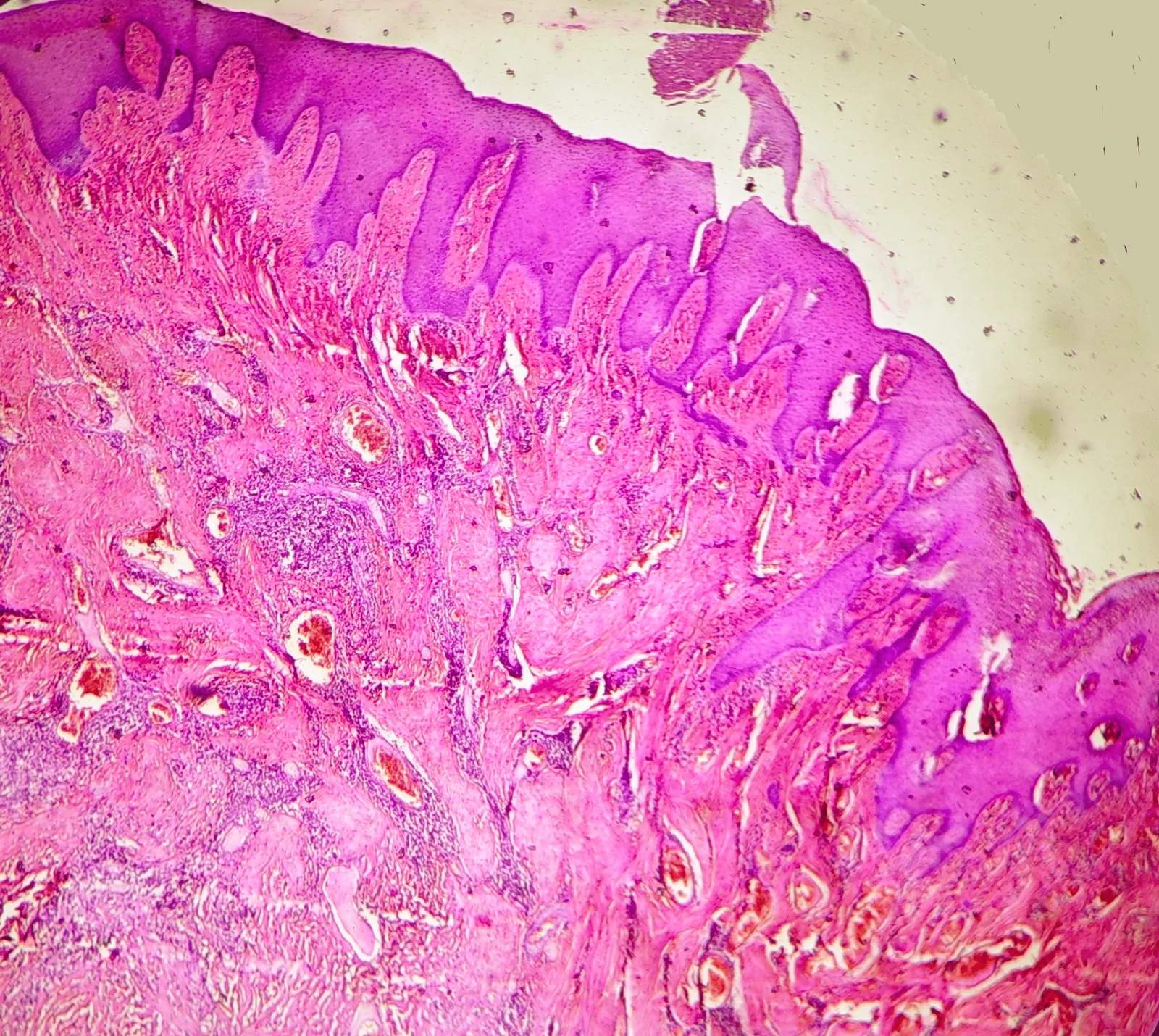
Histopathology of the lesion (10X, H&E).

At the three-month follow-up, the lesion was healed without scarring and with no recurrence (Figure 4).

**Figure 4:**
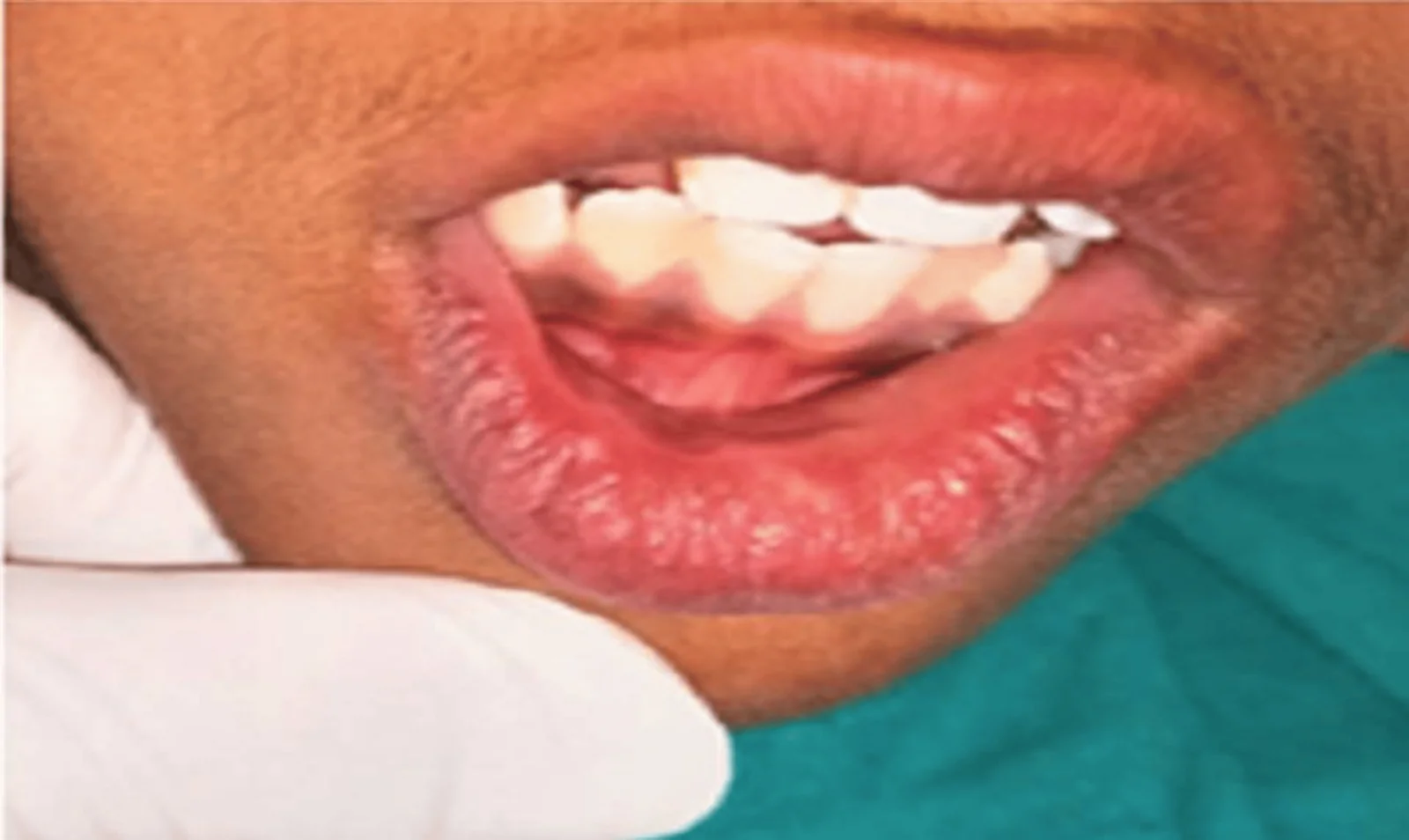
Three-month follow-up.

## Discussion

A literature search across databases, including citation tracking and manual reference screening of the included articles, revealed only 10 reported cases involving the lip in pediatric patients aged up to 14 years (Table 1), with only two cases occurring in the first decade of life.

**Table 1:** Summary of articles included in the review.

Author name	Year	Age/Gender	Site	Size in centimetres	Duration of lesion	History of trauma	Treatment
Pisano M et al [1]	2021	11/F	Lower lip	1.5	˂1 month	Not mentioned	Excision using diode laser
Gonçales ES et al [2]	2010	12/F	Upper lip	1	3 months	absent	Excisional biopsy
Tedjosasongko U et al [3]	2022	10/M	Upper lip	0.7	1 month	present	Surgical excision using electrocautery
Rai A et al [4]	2021	11/M	Lower lip	0.5 x 0.5 x 0.5	2 weeks	present	Surgical blade no. 15, followed by primary tissue closure with 4-0 silk suture
Bin Rubaian NF et al [7]	2021	11/F	Lower lip	1	1 month	present	Shave excision using a blade, followed by electrocautery, silver nitrate, and salt application
Nair PA et al [8]	2016	11/M	Lower lip	3 × 2	2 months	absent	Excision biopsy
Tatar RT et al [9]	2020	12/M	Lower lip	1	Several weeks	present	Excision biopsy
das Chagas MS et al [10]	2009	2 years	Upper lip	0.5 x 0.5	1 month	present	Surgical excision
Al-Qubati Y et al [11]	2014	14/M	Lower lip	1.4	3 months	present	Carbon dioxide snow cautery, fusidic acid topical cream on the lesion twice daily
de Carvalho FK et al [12]	2015	11/M	Upper lip	4.5	25 days	present	Excisional biopsy

Pyogenic granuloma is known by a variety of terminologies, such as granuloma pyogenicum, eruptive hemangioma, granuloma gravidarum, lobular capillary haemangioma, benign vascular tumor, haemangiomatous granuloma, epulis teleangiectatium granulomatosa, and vascular epulis [3,4].

The exact etiology of PG is uncertain; however, several factors are implicated in the etiopathogenesis of PG, but potential contributing and aggravating factors are trauma and hormonal imbalance. A history of systemic medications such as oral contraceptives, retinoids, indinavir, BRAF and EGFR inhibitors, cytogenetic abnormalities, angiogenic factors, overexpression of transcription factors p‑ATF2 and STAT3, gefitinib, cabecitabine, and afatinib is associated with initiation and progression of the lesion [4,8,9]. Trauma could be from chronic irritation due to exfoliation of deciduous teeth, sharp tooth, eruption of permanent teeth, faulty restoration/prosthesis, food impaction, malocclusion, and faulty tooth brushing. It can also be due to self-inflicted soft tissue injuries like lip-biting, cheek biting, and tooth picking. Poor oral hygiene and calculus are important contributing and aggravating factors [4]. All these micro- and macro-trauma trigger an inflammatory response leading to excessive proliferation of vascular connective tissue in an attempt to repair the tissue [2,3]. In the present case, the lesion appears to have developed secondary to trauma, as the patient revealed a history of lip-biting habit.

Various clinical presentations can be noted in PG with some commonly encountered features. The lesion shows a higher prevalence in adults compared to children [10]. It is frequently seen in females during the second, third, and fourth decade of life, attributed to higher levels of estrogen and progesterone hormones in the bloodstream [4]. In the present case, the occurrence was atypical for age as it was present in the first decade of life.

Clinically, it initially appears as a small red papule or nodular lesion which rapidly grows from a few millimeters to centimetres [3,9]. In our patient, the lesion was initially of small size, which increased rapidly in three months to the present size. The colour of PG may vary from reddish-brown to pale pink depending on the degree of inflammatory component superimposition. Initially, the nature of the lesion is predominantly a hyperplastic granulation tissue type with a greater number of immature capillaries; later, the vascularity decreases, and the appearance becomes paler as the collagenous component increases [3]. Based on the clinical course, PG can be categorized into three types: 1. Early lesion: appears pinkish or resembles normal mucosal colour, 2. Established lesion: appears reddish to purplish owing to increased vascularity, and 3. Healing-type lesion: appears pinkish to whitish in colour [4].

The clinical diagnosis of PG should always be confirmed by histological examination of the lesion. PG shows a highly vascular proliferation resembling granulation tissue, which is covered by atrophic or hyperplastic epithelium that may or may not be ulcerated with fibrinous exudates. Its characteristic features show numerous small and large endothelial-lined vascular spaces engorged with red blood cells. Fibroblastic proliferation along with endothelial cell budding and mixed inflammatory cell infiltrate is also evident. These vessels are occasionally organized in lobular aggregates [3,4]. Based on the histopathological features, PG is divided into three phases: 1. cellular, 2. capillary/vascular, and 3. involutionary [4].

Pyogenic granuloma presents rarely in extra-gingival locations. Labial mucosa is an uncommon location than the vermilion of the lip [10]. Since in this case the lesion was present on the lower lip, there was difficulty in speech and poor esthetics due to disfigurement.

Various treatment modalities have been proposed for PG depending on the site and size of the lesion, as well as patient convenience. These include surgical excision, laser surgery, curettage, carbon dioxide snow cryosurgery, shave excision, electrodesiccation, electrocautery, liquid nitrogen cryotherapy, sclerotherapy, topical silver nitrate application, and topical imiquimod therapy [4,8,11,12]. Recently, salt has been reported as a treatment option for PG because it creates a hyperosmolar environment, resulting in lesion shrinkage due to its desiccating effect [4,8,12]. In the present case, laser treatment was considered most appropriate due to lesser age of the patient, less invasive, lesser intra- and postoperative discomfort, and minimal scarring effect.

Recurrence can be attributed to incomplete excision of the lesion, failure to eliminate etiological factors, or repeated trauma to the affected area. Lesions on gingiva show a greater tendency for recurrence than lesions affecting other oral mucosal regions [1]. The lowest rate of recurrence (15.8%) has been reported after conservative excision [4]. In the present case, satisfactory healing was observed with no recurrence at three-month follow-up.

The main advantages offered by laser surgery are high acceptability of lasers in adults as well as in children due to minimal invasiveness as compared to surgical excision, greater patient compliance, reduced use of the anaesthetic, absence of intra- and postoperative discomfort and pain, reduced operating time, effective hemostasis with less intraoperative bleeding, better postoperative management, shorter healing time, and absence of scarring [1].

The differential diagnosis of PG includes peripheral giant cell granuloma, hemangioma, mucocele, traumatic fibroma, Kaposi sarcoma, metastat­ic carcinoma, and other malignant tumors [1,2].

## Conclusions

Extragingival presentation of PG can make clinical diagnosis challenging. A thorough case history and clinical examination are of utmost importance in the diagnosis and treatment planning of the lesion, along with the histopathological examination to establish a final diagnosis. Newer treatment modalities such as laser should be used for children as they are less invasive, reduce intraoperative bleeding, and are better accepted by patients. Elimination of traumatic factors along with regular follow-up may help prevent recurrence of such lesions.
